# Patients with Liver Cirrhosis Show High Immunogenicity upon COVID-19 Vaccination but Develop Premature Deterioration of Antibody Titers

**DOI:** 10.3390/vaccines10030377

**Published:** 2022-02-28

**Authors:** Katharina Willuweit, Alexandra Frey, Moritz Passenberg, Johannes Korth, Nissrin Saka, Olympia E. Anastasiou, Birte Möhlendick, Andreas Schütte, Hartmut Schmidt, Jassin Rashidi-Alavijeh

**Affiliations:** 1 Department of Gastroenterology, Hepatology and Transplant Medicine, University Hospital Essen, University of Duisburg-Essen, Hufelandstr. 55, 45147 Essen, Germany; katharina.willuweit@uk-essen.de (K.W.); alexandra.frey@uk-essen.de (A.F.); moritz.passenberg@uk-essen.de (M.P.); nissrin.saka@uk-essen.de (N.S.); andreas.schuette@uk-essen.de (A.S.); hartmut.schmidt@uk-essen.de (H.S.); 2 Department of Nephrology, University Hospital Essen, University of Duisburg-Essen, Hufelandstr. 55, 45147 Essen, Germany; johannes.korth@uk-essen.de; 3 Institute for Virology, University Hospital Essen, University of Duisburg-Essen, Virchowstr. 179, 45147 Essen, Germany; olympiaevdoxia.anastasiou@uk-essen.de; 4 Institute of Pharmacogenetics, University Hospital Essen, University of Duisburg-Essen, Hufelandstr. 55, 45147 Essen, Germany; birte.moehlendick@uk-essen.de

**Keywords:** SARS-CoV-2, COVID-19, vaccination, liver cirrhosis, end-stage liver disease

## Abstract

SARS-CoV-2 infection is known to lead to severe morbidity and mortality in patients with liver cirrhosis. For this reason, vaccination of these patients against COVID-19 is widely recommended. However, data regarding immunogenicity in patients with liver cirrhosis is limited and even less is known about the kinetics of antibody response, as well as the optimal timing of booster immunization. We analyzed immunogenicity in 110 patients with liver cirrhosis after receiving two doses of the mRNA-based vaccine BNT162b2 following the standard protocol and compared these results to a control group consisting of 80 healthcare workers. One hundred and six patients with liver cirrhosis (96%) developed antibodies against SARS-CoV-2, compared to 79 (99%) in the control group (*p* = 0.400). Still, the median SARS-CoV-2 IgG titer was significantly lower in patients with liver cirrhosis compared to the control group (939 vs. 1905 BAU/mL, *p* = 0.0001). We also analyzed the strength of the antibody response in relation to the time between the second dose and antibody detection. Antibody titers remained relatively stable in the control group while showing a rapid and significant decrease in patients with liver cirrhosis. In conclusion, our data reveals a favorable initial outcome after vaccination with the COVID-19 vaccine BNT162b2 in cirrhotic patients but show a rapid deterioration of the antibody response after time, thereby giving a strong hint towards the importance of early booster immunization for this group of patients.

## 1. Introduction

The messenger RNA (mRNA) COVID-19 vaccine BNT162b2 (Pfizer-BioNTech) has shown excellent efficacy in placebo-controlled trials [1], but specific data regarding the efficacy of COVID-19 vaccination in patients with liver cirrhosis is limited [2]. Despite limited evidence regarding the efficacy of vaccination in this cohort, COVID-19 vaccination is widely recommended for these patients by professional societies, namely the American Association for the Study of Liver Diseases and the European Association for the Study of the Liver [3,4].

Regarding liver transplant recipients, there is an increasing number of published trials showing impaired, but still satisfying, immunogenicity in this group of patients [5,6]. Still, data regarding patients with liver cirrhosis are limited, and, in particular, little is known about the antibody kinetics and durability after vaccination of patients with liver cirrhosis. The evaluation of immunogenicity in these patients is of crucial importance since patients with liver cirrhosis are known to suffer from cirrhosis-associated immune dysfunction, resulting in deficiency of innate and humoral immunity, which predisposes these patients to bacterial and viral infections [7,8]. It is important to note that patients with liver cirrhosis show a high susceptibility to severe courses of COVID-19 [9,10,11], and mortality rates up to 45% were described in patients with decompensated cirrhosis [12]. On the other hand, the response to vaccination in patients with liver cirrhosis is generally impaired in terms of both the strength and the durability of the antibody response after vaccination with different agents [13,14,15,16,17]. Finally, little is known about the longevity of antibody response after COVID-19 vaccination in patients with liver cirrhosis. Given that one of the most pressing contemporary questions about COVID-19 vaccination is when to use booster shots, the durability of vaccination success in this particular cohort is an issue of paramount importance.

We hereby conduct a study in which we analyzed the immune response in 110 patients with liver cirrhosis after vaccination with two doses of the mRNA-based COVID-19 vaccine BNT162b2 (Pfizer-BioNTech). The results were compared to the antibody response of 80 healthcare workers (HCWs) who were also vaccinated using the same protocol.

## 2. Materials and Methods

A total of 110 patients with liver cirrhosis and 80 HCW were vaccinated with the mRNA-based COVID-19 vaccine BNT162b2 (Pfizer-BioNTech, Pfizer Inc., New York, NY, USA, and BioNTech SE, Mainz, Germany) according to the standard protocol with a median time of 42 days (IQR 35–42) between the first and the second dose. Participants under the age of 18, with a previous SARS-CoV-2 infection, or vaccinated with another vaccine were excluded from the analysis. Previous SARS-CoV-2 infections were only diagnosed by self-report, there was no serological screening of possible undiagnosed SARS-CoV-2 infection prior to vaccination. Serum samples were tested for SARS-CoV-2 IgG against the spike glycoprotein using an approved anti-SARS-CoV-2 IgG CLIA (LIAISON^®^ SARS-CoV-2 TrimericS IgG assay, Diasorin, Saluggia, Italy). An arbitrary units per milliliter (AU/mL) ratio of <13.0 was considered to be negative and of ≥13.0 to be positive, according to the manufacturer’s recommendations. A conversion of AU/mL to binding antibody units (BAU/mL), which conforms to the WHO standard, is possible using the following equation: 2.6 × AU/mL = BAU/mL, with 800.0 AU/mL (2080 BAU/mL) being the upper limit of quantification without dilution of the CLIA.

Categorical variables are reported as numbers (percentages) and Fisher’s exact test or the chi-square test were used to compare groups. Quantitative variables are displayed as medians (interquartile ranges, IQR). Nonparametric data were analyzed by the Mann–Whitney U test or the Kruskal–Wallis test to compare groups. A Spearman correlation was used to test correlations between variables. A *p*-value of ≤0.05 was considered to be statistically significant. Statistical analysis was performed with SPSS 27 statistical software (IBM SPSS Statistics; IBM Corporation, Chicago, IL, USA), and GraphPad Prism version 8 for windows (GraphPad Software, San Diego, CA, USA) was used for illustration. This study was conducted according to the guidelines of the Declaration of Helsinki and was approved by the ethics committee of the Medical Faculty of the University of Duisburg-Essen (20-9753-BO and 21-10005-BO).

## 3. Results

### 3.1. Baseline Characteristics

A total of 110 patients with liver cirrhosis followed in our outpatient clinic were enrolled. Approximately 22% of these patients (n = 24) were on the liver transplantation waiting list. Of all cirrhotic patients, 55 were male (50%) and 55 female (50%), whereas the HCW group included less men (n = 20, 25%) than women (n = 60, 75%; *p* = 0.001) (Table 1). The most frequent causes of liver cirrhosis were alcohol consumption (n = 35, 32%) and primary sclerosing cholangitis (n = 18, 16%), followed by autoimmune hepatitis (n = 10, 9%), non-alcoholic steatohepatitis (n = 9, 8%), primary biliary cholangitis (n = 8, 7%), and hepatitis C virus infection (n = 6, 5%). Eight patients (7%) had cryptogenic liver cirrhosis (Table 2). The median model of end stage liver disease (MELD) score at the time of vaccination was 10 (IQR 8–13). Regarding the Child–Pugh classification, 76 patients (69%) were classified as Child A, 31 (28%) as Child B, and 3 (3%) as Child C (Table 1).

The median age at first vaccination was 55 years (IQR 45–61), the median time between the first and second doses was 42 days (IQR 35–42), and median time between the second dose and antibody detection was 69 days (IQR 43–106). Demographic data are depicted in more detail in Table 1.

Regarding the group of HCW, the median age at first vaccination was 54 years (IQR 45–59), the median time between the first and second doses was 44 days (IQR 22–47), and the median time between the second dose and SARS-CoV-2 antibody detection was 56 days (38–90). None of median age, median time between the first and second doses, and the median time between the second dose and antibody detection differed significantly between the two groups.

### 3.2. Antibody Response and Titer after COVID-19 Vaccination

Of all patients with liver cirrhosis, 106 (96%) showed an antibody response after COVID-19 vaccination while four patients (4%) remained negative, whereas 79 (99%) of all HCW turned seropositive with only one patient (1%) lacking a vaccination response (*p* = 0.40) (Table 1). Of the cirrhotic patients without an antibody response, two were male and two were female. Two patients suffered from primary sclerosing cholangitis, one from primary biliary cholangitis, and one from alcohol-induced liver cirrhosis. Concerning Child–Pugh classification, one patient was classified as Child A, two patients as Child B, and one patient as Child C.

The median antibody titer did differ significantly in cirrhotic patients and HCW (939 BAU/mL vs. 1905 BAU/mL, *p* < 0.001). However, stratifying cirrhotic patients according to MELD score (<15; ≥15; *p* = 0.15), Child–Pugh score (Child A; Child B; Child C; *p* = 0.15), or age (<60 years; ≥60 years; *p* = 0.96) did not lead to any significant differences in median SARS-CoV-2 IgG (BAU/mL) levels (Table 3). A Spearman correlation analysis showed no relation/correlation between the MELD score and IgG titer (Spearman coefficient, ρ = −0.066; *p* = 0.49).

### 3.3. Course of Antibody Response in Patients with Liver Cirrhosis and HCW

**Figure 1:**
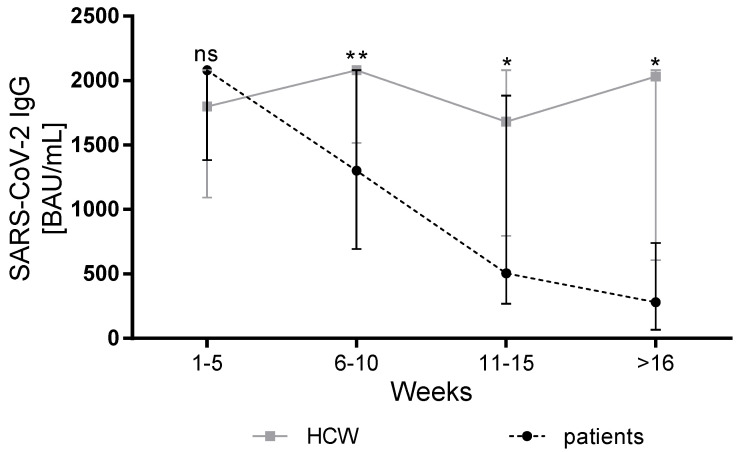
SARS-CoV-2 IgG titer in relation to the time point between the second dose and antibody detection. A comparison of the binding antibody units per milliliter (BAU/mL) ratio of SARS-CoV-2 IgG antibodies of patients with liver cirrhosis and HCWs at different times after the second dose. Patients and HCWs were grouped based on the weeks between the second dose and antibody detection. The medians are illustrated with the corresponding interquartile ranges. BAU: binding antibody units; HCW: healthcare worker; SARS-CoV-2: severe acute respiratory syndrome coronavirus type 2; ns: no significance, * *p* ≤ 0.05; ** *p* ≤ 0.01.

In order to analyze the dependence of antibody levels on the time between antibody detection and vaccination, we grouped both patients and HCW into different groups based on the time of antibody detection and compared the median antibody titer between both groups. For both patients and HCW, similar median antibody titers were observed in the first period after vaccination (week 1–5) (liver cirrhosis patients: n = 22, median 2080 BAU/mL; HCW: n = 19; median 1720 BAU/mL (IQR 1070 to >2080) (*p* = 0.21). For all other periods, we observed consistently lower antibody titers in cirrhotic patients compared to HCW (week 6–10: patients n = 37, median 1300 BAU/mL vs. HCW n = 22, median 2080 BAU/mL, *p* < 0.01; week 11–15: patients n = 23, median 570 BAU/mL vs. HCW n = 31, median 1680 BAU/mL, *p* = 0.01; ≥16 weeks: patients n = 28, median 263 BAU/mL vs. HCW n = 7, median 2030 BAU/mL, *p* = 0.01) (Figure 1). Overall, the antibody titers in the HCW group remained relatively stable, while the kinetics in the group of patients with liver cirrhosis showed a rapid decrease depending on the time between vaccination and antibody detection.

## 4. Discussion

This study describes the immunogenicity of 110 patients with known liver cirrhosis after standard protocol-based vaccination of two doses of the mRNA-based COVID-19 vaccine BNT162b2 (Pfizer-BioNTech) in comparison to a healthy control group. The rate of patients who elicited antibody reaction after vaccination was encouragingly high (96%) and did not show significant differences to the control group. However, the value of SARS-CoV-2 IgG titers was significantly lower in the study group compared to the control group (*p* < 0.0001). Most importantly, the SARS-CoV-2 IgG value depended on the time between the second dose and antibody detection, showing a more rapid decline in the group of patients with liver cirrhosis compared to the group of HCW.

Patients with liver cirrhosis are known to have a considerably high vulnerability regarding SARS-CoV-2 infection, with high mortality rates, and vaccination is of crucial importance [9,10,11,12,18]. Accordingly, clinical data show that the risk of hospitalization in immunized patients with liver cirrhosis is clearly reduced [19]. Other studies analyzing immunogenicity in patients with impaired liver function showed similar results. In patients with non-alcoholic fatty liver disease (NAFLD), seropositivity rates of 95.5% were described [20]. In patients with liver cirrhosis, Thuluvath et al. reported a seroconversion of 95.9% in a group of 79 patients with liver cirrhosis [21], and Calleri et al. observed a seroconversion rate of 94.4% in 89 pre-transplant patients [22].

SARS-CoV-2 IgG titers happened to be significantly lower in our cohort compared to the group of HCW. This is an important point, although its relevance in terms of humoral immunity is not yet entirely clear, since exact antibody thresholds conferring humoral immunity are still vague [23,24,25] and investigations in this direction are ongoing.

The most interesting point of this study refers to the kinetics of the antibody titers. In previous reports in healthy participants, antibody titers stayed relatively stable in the first months after vaccination, although a certain decay was observed [26,27,28,29,30,31]. In our cohort, the dynamics of antibody titers also showed relative stability in the group of HCW. In contrast, the values of SARS-CoV-2 antibody titers differed depending on the time interval between the second dose and antibody detection, showing a significantly more rapid reduction in antibody titers in the group of cirrhotic patients than in the control group. This goes in line with the previous studies concerning the reduced durability of the antibody response upon vaccination of patients with cirrhosis with other vaccines [14,15,17,32]. With the question of the optimal timing of booster immunization remaining unanswered, our data indicate that an early application of booster vaccination in cirrhotic patients is crucial. Of course, our results do not provide specific instructions regarding the exact timing of booster immunization. To answer this question, more data, especially related to the longitudinal assessment of antibody titers and the analysis of the severity of SARS-CoV-2 infections after COVID-19 vaccination in cirrhotic patients are needed. Longitudinal data will help determine whether antibody responses show an early decline after booster immunization as well and if additional doses are needed. Still, since booster immunization is highly effective in the reduction of mortality [31] and also helps protect against the omicron variant [32,33], the premature deterioration of antibody response in our cohort raises awareness of the importance of booster immunization in this vulnerable cohort.

Reduced longevity of the antibody response after vaccination in cirrhotic patients is understandable, since these patients are known to suffer from complex alterations of the immune system, namely cirrhosis-associated immune dysfunction [7,34]; in this regard, a profound reduction in peripheral B cell counts and the dysfunction of memory B cells in cirrhotic patients are reported [35,36,37]. In addition, T-cell deficiency is common in cirrhotic patients, even in the early stages of cirrhosis, also affecting T helper cells, which are essential for maintaining durable immune responses [38,39,40]. In this context, reduced T-cell response rates after COVID-19 vaccination in cirrhotic patients compared to healthy controls have been reported [41].

Nevertheless, our study, of course, has limitations. The study was performed on a monocentric cohort, the time between vaccination and antibody detection varies, and sex distribution differs between the study group and our control group. Different confounding factors, such as the possible intake of immunosuppressive medication or nicotine consumption, which are reported to have an impact on antibody response and durability after vaccination [42], have not been taken into account in this study. Additional characterization of the immune response, such as analyzing the memory B-cell and T-cell-mediated immune reaction or the neutralizing capacity of antibodies, could be of interest and are not represented in this study. Since SARS-CoV-2 infections prior to vaccination were only diagnosed by self-report without serological screening, undiagnosed pre-vaccination infection and possible immune priming effects of these infections cannot be ruled out. In addition, most of our patients had Child–Pugh class A or class B cirrhosis, with only three patients with Child C cirrhosis. Still, we have an otherwise homogenous, relatively large cohort with all patients suffering from liver cirrhosis and all of them were vaccinated with the same vaccine.

We are convinced that the results of our study have important clinical implications and are meaningful for the vulnerable cohort of patients with liver cirrhosis. The study presents satisfying initial results in terms of immunogenicity but also gives an important alert regarding potential premature deterioration of antibody titers and, thereby, indicates there are potential beneficial effects of early booster immunization. Further investigations, particularly in the longitudinal assessment of the course of antibody titers and potential breakthrough infections, will be highly relevant to assess an efficient vaccination strategy for durable humoral immunity in patients with liver cirrhosis.

## Figures and Tables

**Table 1:** Patient characteristics are presented as absolute number, n, and percentage or as median and interquartile range.

Patient Characteristics	Patients n/(%)	HCW n/(%)	*p*-Value
Total patient number	110	80	-
Sex (male/female)	50 (50)/55 (50)	20 (25)/60 (75)	0.001
Child A/Child B/Child C	76 (69)/31 (28)/3 (3)	-	
SARS-CoV-2 IgG detectability rate	106 (96)	79 (99)	0.40
	Median (IQR)	Median (IQR)	
MELD score at first vaccination	10 (8–13)	-	-
Age at first vaccination [years]	55 (45–61)	54 (45–59)	0.19
Time between first and second doses [days]	42 (35–42)	44 (22–47)	0.39
Time between second dose and SARS-CoV-2 Ab detection [days]	69 (43–106)	56 (38–90)	0.20
SARS-CoV-2 IgG (BAU/mL)	939 (307 to >2080)	1905 (996.3 to >2080)	0.0001

Ab: antibody, BAU: binding antibody units, HCW: healthcare workers, IQR: interquartile range, MELD: model of end-stage liver disease; SARS-CoV-2: severe acute respiratory syndrome coronavirus type 2.

**Table 2:** Etiology of liver cirrhosis is shown as absolute number, n, and percentage.

Diagnosis	n (%)
Alcoholic-induced liver cirrhosis	35 (32)
Primary sclerosing cholangitis	18 (16)
Autoimmune hepatitis	10 (9)
Non-alcoholic steatohepatitis	9 (8)
Primary biliary cholangitis	8 (7)
Cryptogenic liver cirrhosis	8 (7)
Hepatitis C virus-induced liver cirrhosis	6 (5)
Secondary sclerosing cholangitis	5 (5)
Hepatitis B virus-induced liver cirrhosis	3 (3)
Wilson’s disease	3 (3)
Hepatocellular carcinoma	1 (1)
Others ^1^	4 (4)

^1^ Others: Bile duct atresia, Joubert syndrome, Gaucher’s disease, Budd-Chiari syndrome (n = 1 each).

**Table 3:** Comparison of the antibody response in different groups of patients with liver cirrhosis. Patients grouped by age, MELD score, and class of Child–Pugh classification.

Patients	SARS-CoV-2 IgG (BAU/mL) Median (IQR)	*p*-Value
Age		
<60 years (n = 76)	965 (325.5 to >2080)	0.96
≥60 years (n = 34)	740 (293 to >2080)	
MELD score		
<15 (n = 97)	965 (344.5 to >2080)	0.15
≥15 (n = 13)	570 (128.45–1680)	
Child score		
Child A (n = 76)	968 (362.5 to >2080)	0.15
Child B (n = 31)	815 (203 to >2080)	
Child C (n = 3)	203	

HCW: healthcare worker; IQR: Interquartile range; MELD: model of end-stage liver disease; SARS-CoV-2: severe acute respiratory syndrome coronavirus type 2.

## Data Availability

The data that support the findings of this study are available from the corresponding author upon reasonable request.
